# Right-sided colopleural fistula secondary to diverticular disease: a case report

**DOI:** 10.1186/s13256-022-03668-1

**Published:** 2022-11-27

**Authors:** Summer Hassan, Primal Singh

**Affiliations:** 1 Department of General Surgery, Middlemore Hospital, Auckland University, 100 Hospital Road, Auckland, New Zealand; 2 University of Auckland, Auckland, New Zealand

**Keywords:** Colopleural fistula, Diverticular disease, Right hemicolectomy, Colorectal fistula, Case report

## Abstract

**Background:**

Colopleural fistulas are mostly left-sided and related to trauma, Crohn’s disease, or gastrointestinal malignancy. However, a diverticular fistula between the colon and right pleural space has not been reported and is rare considering the liver forms a natural anatomical barrier on this side. Colopleural fistulas often present with respiratory symptoms ranging from mild cough and dyspnea to sepsis from empyema caused by the leakage of gastrointestinal content into the pleural space. Although colopleural fistulas are rare, maintaining low suspicion is pivotal for timely investigation and appropriate surgical planning, particularly in the context of previous intra-abdominal infections or trauma.

**Case presentation:**

A 67-year-old Chinese male presenting with prolonged respiratory symptoms was found to have a right-sided colopleural fistula confirmed by computed tomography imaging and a colonoscopy. It was addressed surgically after multidisciplinary consensus was reached, with a right hemicolectomy and repair of the diaphragmatic defect. The patient recovered remarkably well with resolution of respiratory symptoms.

**Conclusion:**

Appropriate work-up of a suspected colopleural fistula with radiological and endoscopic investigations to determine anatomy and etiology is crucial. Most cases will require surgical management, and involvement of the respiratory and cardiothoracic teams is important to optimize lung function preoperatively and plan for possible chest complications.

## Background

Fistulas are formed by the abnormal communication between two or more epithelial surfaces. Colopleural fistulas are formed between the colon and the pleura. These are mostly left sided and caused by trauma, previous abdominal surgery, Crohn’s disease, or gastrointestinal malignancy. However, a diverticular fistula between the colon and right pleural space is rare considering the liver forms a natural anatomical barrier on this side. Patients with colopleural fistulas often present with varying degrees of respiratory symptoms, including shortness of breath, chronic cough, and in some cases can suffer from empyema and ensuing sepsis. In patients with previous intra-abdominal trauma, surgery, infections, or bowel disease, keeping a low suspicion is paramount for prompt imaging and interrogation of the fistula tracts. Surgical management of these fistulas can pose a challenge depending on the anatomical location and the complexity of the tracts formed, prompting potential involvement of multiple surgical services, for example, colorectal, upper gastrointestinal tract, and cardiothoracic surgeons. Therefore, early recognition is important to ensure timely surgical planning. This report presents a rare case of right-sided colopleural fistula secondary to diverticular disease and sheds light on the patient’s symptoms and investigational modalities that facilitated the diagnosis and the treatment approach.

## Case presentation

**Fig. 1:**
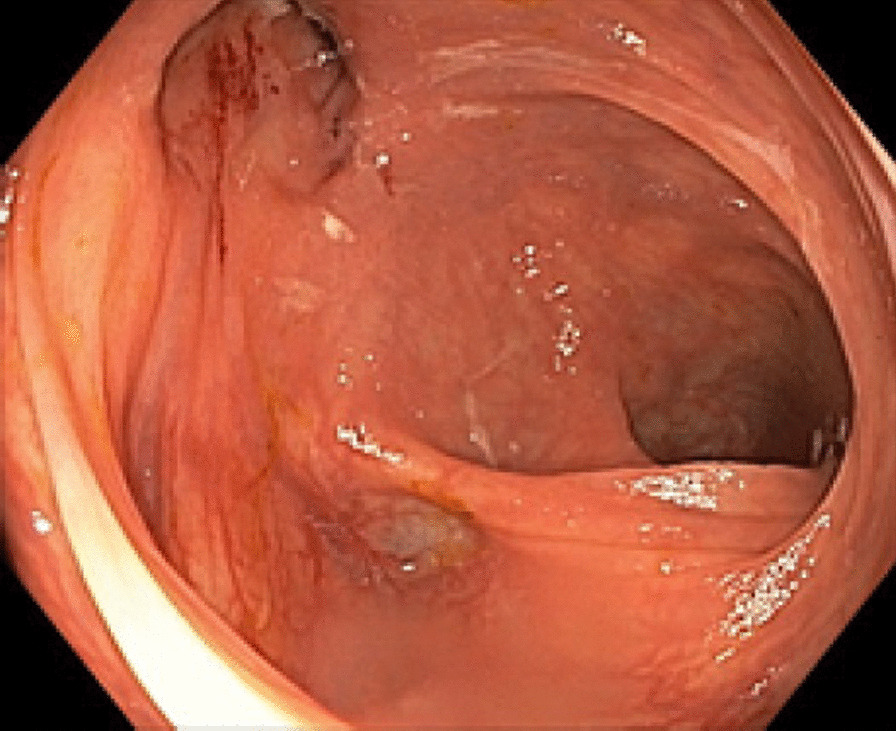
Colonoscopy image showing two diverticula at the hepatic flexure

**Fig. 2:**
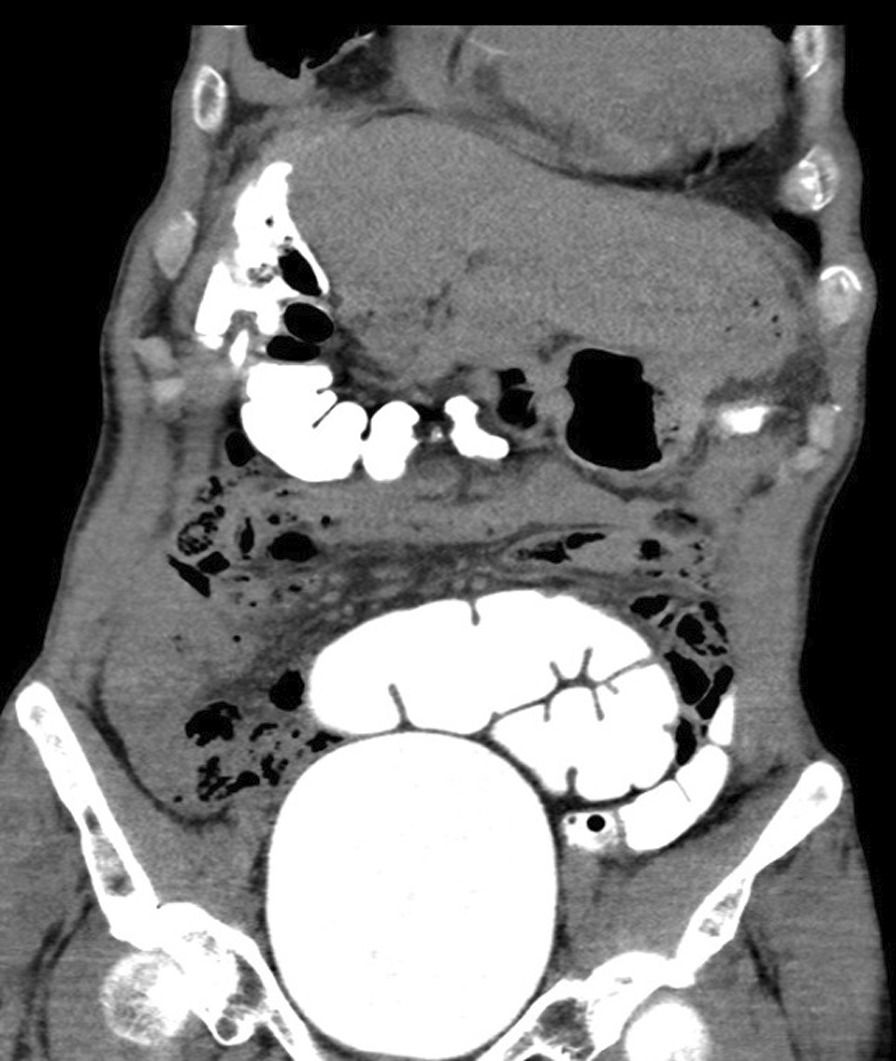
Computed tomography (CT) image confirming a colopleural fistula with contrast outlining a fistula tract between the colon and right lower lobe

A 67-year-old Chinese male presented to the respiratory clinic with several months of a productive cough and exertional dyspnea with no abdominal symptoms. He had a background of chronic hepatitis B, pulmonary tuberculosis treated in 2002, and an upper midline laparotomy in China 20 years prior for a perforated gastric ulcer. Computed tomography (CT) of chest showed mild bronchiectasis and multifocal consolidation in both lower zones with gas tracking from the colonic hepatic flexure to the right pleural cavity, suggesting a colopleural fistula. Bronchoscopy showed no obstructing endobronchial lesion, and bronchial washings grew *Escherichia coli* and mixed anaerobes with no mycobacterium and no malignant cells. Colonoscopy showed two diverticula at the hepatic flexure, one particularly large (Fig. 1—colonoscopy). CT scan of the abdomen with rectal contrast confirmed a colopleural fistula with contrast outlining a fistula tract between the colon and right lower lobe (Fig. 2—CT).

**Fig. 3:**
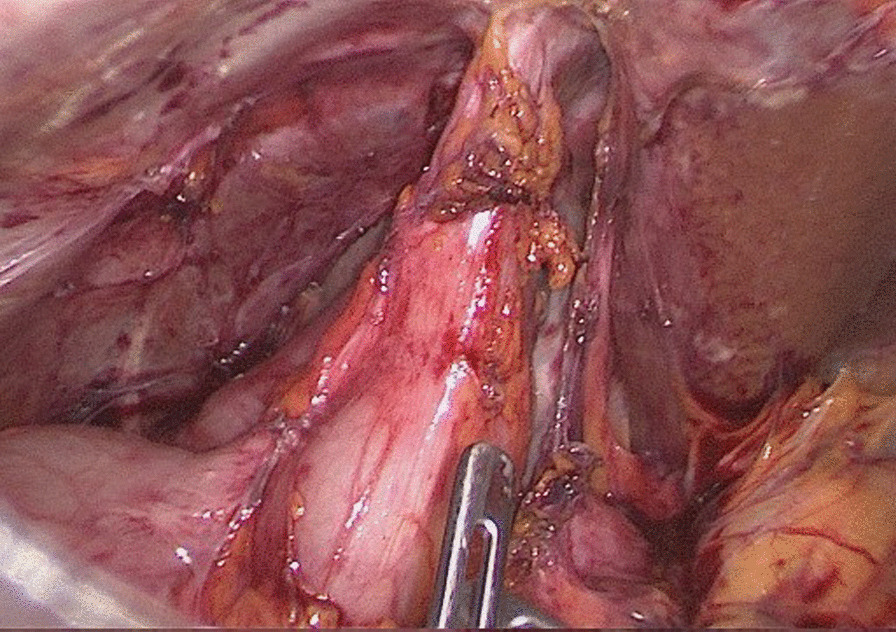
Laparscopic image showing the adhesions involving the liver to the diaphragm, the omentum, distal stomach, and a short segment of colon

Following discussion with the respiratory and cardiothoracic teams, he was arranged to undergo resection of the involved colonic segment and repair of the associated diaphragmatic defect, then insertion of a right chest drain for an expected postoperative pleural effusion. Preoperative preparation included lung function tests, which were normal, chest physiotherapy to establish good airway clearance, two weeks of oral amoxicillin/clavulanate to reduce the microbial load in the respiratory tract, and bowel preparation the day before the operation. Laparoscopy showed the liver was adherent to the diaphragm in the right upper quadrant along with the omentum, distal stomach, and a short segment of colon also adherent just anterior to the liver edge. Adhesions were divided laparoscopically to isolate the segment of colon leading up to the diaphragm with the stomach, gallbladder, and liver safely dissected away (Fig. 3—laparoscopic). He developed mild subcutaneous emphysema related to the laparoscopic insufflation and diaphragmatic defect from the fistula; therefore, a chest drain was inserted into the right pleural cavity with palpation showing the right lung to be tethered basally. The right colon was mobilized laparoscopically with the ileocolic and right colic vessels ligated, achieving good colonic mobility and leaving only the short segment tethered to the diaphragm. A right subcostal incision was made, allowing this segment of colon to be dissected off the diaphragm. A 2 cm diaphragmatic defect at the site of the fistula was repaired with interrupted 2/0 Ethibond sutures. A right hemicolectomy was performed with a stapled side-to-side ileocolic anastomosis. An omental flap was placed over the diaphragmatic repair. The patient had an uneventful postoperative course with resolution of respiratory symptoms at 3-month follow-up. Histology of the colon showed inflammation and scarring in keeping with a fistula.

## Discussion and conclusions

Colopleural fistulas are a rare complication mostly attributed to trauma, iatrogenic injury, Crohn’s disease, or gastrointestinal malignancy [1–4]. Fistulization can also complicate diverticular disease, affecting 4–20% of these patients [5]. Diverticular fistulas tend to arise from the left side, connecting the large bowel with surrounding structures, such as the small bowel, the urinary bladder, the genitalia, the skin, and in rare cases, the pleural space [6, 7]. Right-sided diverticular disease is less frequent and—as in this case—tends to affect patients of Asian ethnicity [8]. Right-sided colopleural fistulization has not been reported in the literature, which is likely owing to the anatomical barrier formed by the liver, making such presentation very unusual. The possible cause of the fistula in this patient may be related to adhesions following his previous laparotomy for a perforated gastric ulcer, causing a segment of the right colon to become tethered to the right diaphragm and later development of a diverticulum within this segment with associated perforation leading to a fistula. Appropriate investigation involved a CT scan with rectal contrast, which was successful in outlining the fistula tract and localizing it, and colonoscopy enabled direct visualization of the diverticula. Furthermore, bronchoscopy was useful in confirming the absence of an obstructing endobronchial lesion, as well as obtaining samples that grew enteric microbes. This provided further confirmation of the diagnosis and influenced the choice of antibiotics. The definitive management of colopleural fistulas is surgical. As described in this case, the surgical intervention consists of resecting the involved segment of bowel and aiming for primary anastomosis, unless infected inflamed tissue at the anastomotic region precludes this. When feasible, the colonic anastomosis should be separated from the area of inflammation using an omental patch and finally repairing the diaphragmatic defect. The decision to place a chest drain should be considered, and in this case, it was guided by concern for pneumothorax secondary to the laparoscopic insufflation.

Although diverticulitis as a cause of colopleural fistulization is uncommon, a few similar cases have been described in the literature, reporting patients presenting with respiratory symptoms that evolve to pleural effusion and empyema. In these cases—as in our case—the growth of gut microbes from pulmonary samples raised the suspicion of potential colopleural fistulization. This was then followed by imaging modalities, with CT scans being the most frequently used, as well as a colonoscopy and sinograms [1, 2, 7, 9–11]. There is agreement across these cases that surgical repair of these fistulas is the management of choice. The distinctive difference between this case and similar published reports is the unexpected location of the fistulization on the right side, highlighting the importance of keeping a low threshold of suspicion for accurate timely diagnosis.

Learning points from this case include appropriate work-up of a suspected colopleural fistula with radiological and endoscopic investigations to determine anatomy and etiology. Most cases will require surgical management, and involvement of the respiratory and cardiothoracic teams is important to optimize lung function preoperatively and plan for possible chest complications, such as a postoperative effusion or empyema, or a concurrent lung resection in more severe cases.

## Data Availability

All the information provided in this manuscript is supported by clinical notes, formal reports, and images from the radiographic investigation modalities and clinical letters.
